# Transcutaneous Electrical Nerve Stimulation Regulates Organ Blood Flow and Apoptosis during Controlled Hypotension in Dogs

**DOI:** 10.1371/journal.pone.0094368

**Published:** 2014-04-14

**Authors:** Lele Zhang, Xiaomei Shao, Chuanlong Zhou, Xiaoqing Guo, Ling Jin, Linli Lian, Xiaojing Yu, Zhenhua Dong, Yadi Mo, Jianqiao Fang

**Affiliations:** Department of Neurobiology and Acupuncture Research, Third Clinical Medical College, Zhejiang Chinese Medical University, Hangzhou, Zhejiang Province, People's Republic of China; Glasgow University, United Kingdom

## Abstract

Transcutaneous electrical nerve stimulation (TENS) is commonly used in clinical practice for alleviating pains and physiological disorders. It has been reported that TENS could counteract the ischemic injury happened in some vital organs. To determine the protective effect of TENS on internal organs during CH in dogs, target hypotension was maintained for 60 min at 50% of the baseline mean arterial pressure (MAP). The perfusion to the brain, liver, stomach, and kidney was recorded and apoptosis within these organs was observed. Results showed that when arriving at the target MAP, and during the maintaining stage for 10 min, perfusion to the stomach and liver in the CH+TENS group was much higher than in the CH group (*P*<0.05). Perfusion to the cerebral cortex greatly declined in both the controlled pressure groups when compared with the general anesthesia (GA) group (*P*<0.05). After withdrawing CH, the hepatic blood flow in both the CH and CH+TENS groups, and the gastric and cerebral cortical blood flow in the CH+TENS group, were rapidly increased. By the end of MAP restoration, gastric blood flow in the CH group was still low. At 72 h after applying CH, terminal deoxynucleotidyl transferase-mediated dUTP-biotin nick end labeling (TUNEL)-positive cells in stomach and kidney tissue from the CH group were significantly increased compared with those in the GA group (*P*<0.05). There was no significant difference in TUNEL-positive cells in the liver and hippocampus among the three groups. Our results demonstrated that CH with a 50% MAP level could cause lower perfusion to the liver, stomach, cerebral cortex, and kidney, with apoptosis subsequently occurring in the stomach and kidney. TENS combined GA is able to improve the blood flow to the liver, stomach, and reduce the apoptosis in the stomach and kidney.

## Introduction

Controlled hypotension (CH) is frequently used in major surgeries [1], [2]. The primary advantages of this technique are minimization of surgical blood loss, better wound visualization, reduced duration of surgery, and increasing security of surgery [3], [4]. However, owing to the low perfusion secondary to CH, ischemic injury may occur in vital organs (such as the brain and kidney) [5].

Transcutaneous electrical nerve stimulation (TENS) is an external method for pain and hypotension relief [6]–[9]. Several studies demonstrate that the possible mechanisms underlying TENS analgesia may be related to the endogenous opioid system [10], [11]. Similar neurochemical mechanisms, transduction pathways, and regulation are seen with TENS and electrical acupuncture stimulation [electroacupuncture (EA)], a Chinese medical therapy, when used for analgesia [12], [13]. Previous studies revealed that TENS has become a novel complementary therapy and an alternative to conventional medicine in clinical practice because of its safety and lack of adverse reactions [14], [15]. Accumulating clinical and experimental evidence has demonstrated that TENS and electrical acupuncture stimulation can effectively increase local blood flow and blood perfusion to the stomach and reduce intestinal ischemia [16]–[18]. Additionally, it also can increase blood perfusion to the ovary by modulating central circulatory systems [19]. In our previous studies, we also found that TENS combined general anesthesia (GA) could enhance blood perfusion to the liver when used with controlled hypotension at a 60% mean arterial pressure (MAP) level [20]. This increased perfusion also occurred in the thalamus at a 30% MAP level [21]. By contrast, previous studies also showed that electrical stimulation protected the nigrostriatal system via multiple mechanisms including antioxidation and antiapoptosis [22], [23]. Other relevant studies found TENS could reduce apoptosis and regulate apoptosis-controlling genes [24]. Additionally, in our previous research, we also found TENS combined GA for CH with 60% and 30% MAP levels could effectively inhibit the occurrence of apoptotic cells in the liver and heart [20], [25].

In view of this, it is necessary to determine whether TENS combined GA has a similar active role in the protection of vital organs under CH with a 50% MAP level. This study used a canine model to determine the changes in organ blood flow and apoptosis in vital organs under or after CH (50% level of baseline MAP) alone or in combination with TENS, to provide experimental evidence for the clinical application of TENS-assisted anesthesia in cases using CH.

## Materials and Methods

### Ethics statement

All animal procedures performed in this work followed guidelines in accordance with the Regulations for the Administration of Affairs Concerning Experimental Animals, and were approved by the Animal Care and Welfare Committee of Zhejiang Chinese Medical University, Zhejiang, China (Protocol number of Animal Experimental Ethical Inspection 201096).

### Animals

Male beagle dogs weighing approximately 9–12 kg were supplied by the Laboratory Animal Center of Zhejiang Chinese Medical University, Zhejiang, China. Animals were housed individually in an artificially lit, temperature-controlled room (23±1°C) with a fixed 12-h on/off light cycle. Food and water were available ad libitum in the home cage. Dogs were allowed at least 3 days to acclimate to their home environment before experiments. Eighteen beagle dogs were randomly divided into three groups: the GA group, the GA-induced CH group (CH group), and the combined TENS group with GA-induced CH (CH+TENS group).

### TENS stimulation

**Figure 1:**
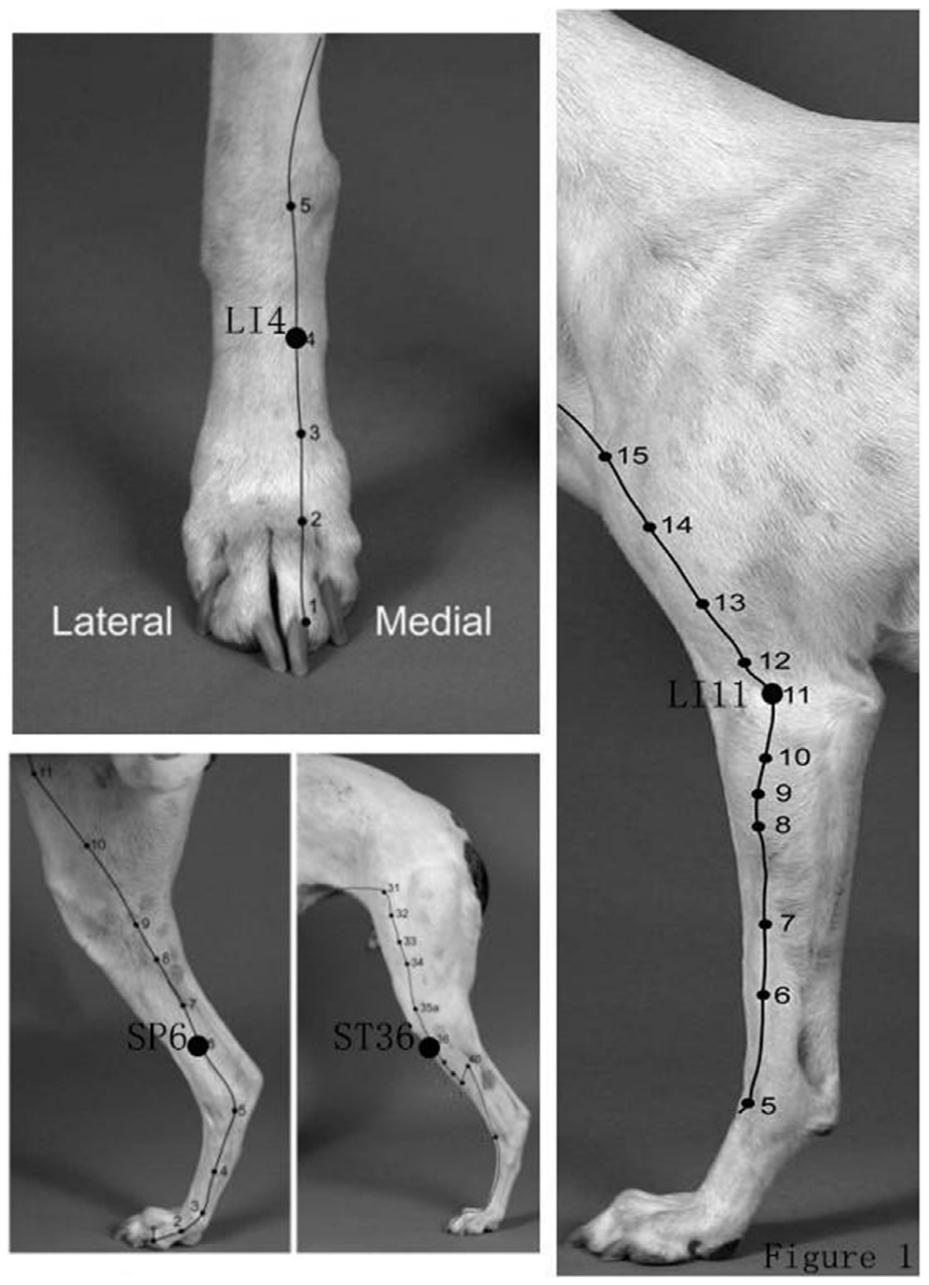
The location of all four acupuncture points. *Hegu* (LI 4) is on the medial side of the thoracic limb between the second and third metacarpal bones at the midpoint of the third metacarpal bone. *Quchi* (LI 11) is on the lateral side of the thoracic limb at the lateral end of the cubital crease, halfway between the lateral epicondyle of the humerus and the biceps tendon with the elbow flexed. *Sanyinjiao* (SP 6) is On the medial side of the pelvic limb 3 cun proximal to the tip of the medial malleolus in a small depression on the caudal border of the tibia (opposite GB-39 on the lateral side). *Zusanli* (ST 36) is on the craniolateral aspect of the pelvic limb, 3 cun distal to ST-35, 0.5 cun lateral to the cranial aspect of the tibial crest, in the belly of the cranial tibialis muscle; this is a long linear point.

In the CH+TENS group, eight square self-adhesive electrodes (5×5 mm) were applied at bilateral acupoints Hegu (LI 4), Zusanli (ST 36), Sanyinjiao (SP 6), and Quchi (LI 11). The transpositional method was used to determine the acupoints in dogs. In this method of *the book of “Xie's Veterinary Acupuncture (edited by Xie HS, Preast V, Ames, Iowa)”* (LI 4 in Page 138, ST 36 in Page 147–148, SP 6 in Page 151, LI 11 in Page 139–140) (Figure 1), the veterinary acupoints were located by transforming human acupoints onto the animal anatomy [26]. Bidirectional rectangle electrical pulses at 0.2 ms width (in 100 Hz) or 0.6 ms width (in 2 Hz), with intensities ranging from 3 to 5 mA (designated as the CH+TENS group) were given for a total of 30 min before CH. The TENS group was stimulated from this time to the end of the maintenance stage.

### Experimental protocol

For simplicity, all time points involved in the study were described as follows, after necessary anesthesia procedures (T_1_), before CH (T_2_), reaching target MAP (T_3_), maintaining target MAP for 10 min (T_4_), maintaining target MAP for 30 min (T_5_), maintaining target MAP for 60 min (T_6_), increasing MAP for 10 min (T_7_), increasing MAP for 30 min (T_8_), increasing MAP for 60 min (T_9_), and end of MAP increase (T_10_). Furthermore, the duration of time from T_1_ to T_2_ was 30 min.

Dogs in the CH and CH+TENS groups were administered the same inhalational anesthesia (isoflurane) and intravenous anesthesia using sodium nitroprusside (SNP) for CH. MAP was maintained in the control pressure groups within the limits of 50–60 mmHg for 60 min. Dogs in the GA group were only anesthetized with isoflurane without inducing CH.

All dogs had been fasted for 12 h before the beginning of experiments. Within 30 min before intravenous anesthesia, intramuscular injection of atropine sulfate occurred. When radial intravenous injections of propofol (5 mg/dog•10 s) and vecuronium (0.1 mg/kg) had been applied for inducing anesthesia and maintaining muscle relaxation, dogs were fully anesthetized. All animals then underwent endotracheal intubation and mechanical ventilation. Throughout the protocol, the respiratory rate was controlled at 15–18 breaths per minute and the tidal volume at a range of 180–230 ml, to ensure arterial blood gas pH values in the range of 7.35–7.40 and maintain normoxia and normocarbia (PaCO_2_ = 35–45 mmHg). Isoflurane (1.5%) was administered for maintaining anesthesia in the GA group. The isoflurane concentration was adjusted to 3% from the beginning of CH. After 5 ml of isotonic saline was given intravenously followed by continuous infusion (6 ml/h/kg), animals were allowed to stabilize for 5 min. Thirty minutes before the performance of CH, intravenous injection of vecuronium (0.05 mg/kg) occurred to prevent animals spontaneously breathing during CH. We then placed an indwelling venous catheter in the left jugular vein for the infusion of SNP (1 µg/kg) for CH. The initial concentration of SNP was 0.1 mg/ml and increased at the rate of 1 µg/kg/min until the target MAP level was reached. The target low MAP was maintained for 60 min, and after this maintenance stage, the infusion of SNP was stopped. Isoflurane concentration was also adjusted to 1.5%. As the MAP returned to baseline, all blood pressure controlling equipment was withdrawn, and routine surgical care was given after the application of CH. After then, all dogs were back to be housed individually in the temperature-controlled room (23±1°C) with a fixed 12-h on/off light cycle. Food and water were also available ad libitum in the home cage for 72 h. At 12 h after CH, all dogs were administrated by intramuscular injection of penicillin, however, analgesics were not performed for the dogs of any group.

### The measurement of canine femoral artery MAP

When dogs were anesthetized, routine disinfection in the right femoral artery area was performed, and then a 2-cm incision on the skin area of the right femoral artery was created. The femoral artery catheter that connected to the biological signal acquisition system (RM6240BD, China) was immediately placed so as to obtain continuous real-time monitoring of canine femoral arterial MAP.

### The measurement of blood flow to the cerebral cortex, liver, kidney, and stomach

After MAP measurement, the head of the animal was fixed into a stereotaxic instrument and the top of the head sterilized. The local skin was then cut and blunt dissection of the muscles of the skull surface occurred. A hole (diameter approximately 5 mm) was punched into the skull using a cranial drill, in which the probes of a Laser Doppler blood flow meter (PeriFlux system 5000, Sweden) was placed close to the cerebral cortex surface for continuous dynamic monitoring of cerebral cortical blood flow. After this manipulation, we sterilized the surface skin of the projection area of each vital organ to be studied, created a 3-cm incision along the direction of the muscle bundle, and blunt dissected the muscles until the liver, kidney, and stomach were exposed to view. Laser Doppler blood flow meter probes were then placed on the surface of the liver, kidney, and stomach, and fixed into place with a glue of Loctite 4161 and an accelerator of Bsi Bob smith industries.

It should be noted that all of the above operations were performed in a sterile environment and surgical practice was strictly adhered to during invasive procedures.

### TUNEL staining

At 72 h after CH, animals were sacrificed by intraperitoneal injection of chloral hydrate solution, and then segments of the hippocampus, liver, kidney, and stomach were harvested and fixed in 4% paraformaldehyde and used for histological examination. All tissues were dehydrated in ethanol and embedded in paraffin. Terminal deoxynucleotidyl transferase-mediated dUTP-biotin nick end labeling (TUNEL) assay was performed according to the manufacturer's instructions (In Situ Cell Death Detection Kit; Roche, Germany). Briefly, paraffin-embedded sections were deparaffinized with xylene followed by absolute ethanol, and decreasing concentrations of ethanol (95% through to 70% ethanol). Sections were washed with phosphate-buffered saline (PBS) for 5 min and treated with protease K (20 mg/mL) for 30 min at room temperature. After washing in PBS twice, these sections were incubated with 50 ml of TUNEL reaction solution in a humidified environment at 37°C for 60 min. Following washing in PBS (three times), these sections were treated with 50 ml of converter-POD in a humidified environment at 37°C for 30 min. Following another washing step (three times in PBS), sections were incubated with 100 µl of substrate at room temperature for 10 min. Sections were mounted after washing in PBS (as above). The number of TUNEL-positive cells was counted under a microscope (40×) in five independent fields and then averaged.

### Data analysis

Values were expressed as mean ± standard error of the mean. All statistics were performed using SPSS version 13.0 for Windows. Differences between means were analyzed using Two- way ANOVA with repeated measures analysis of variance followed by the Newman–Keuls post-hoc test of differences between means. A *P* value of less than 0.05 was considered statistically significant.

## Results

### Changes in MAP under CH

**Figure 2:**
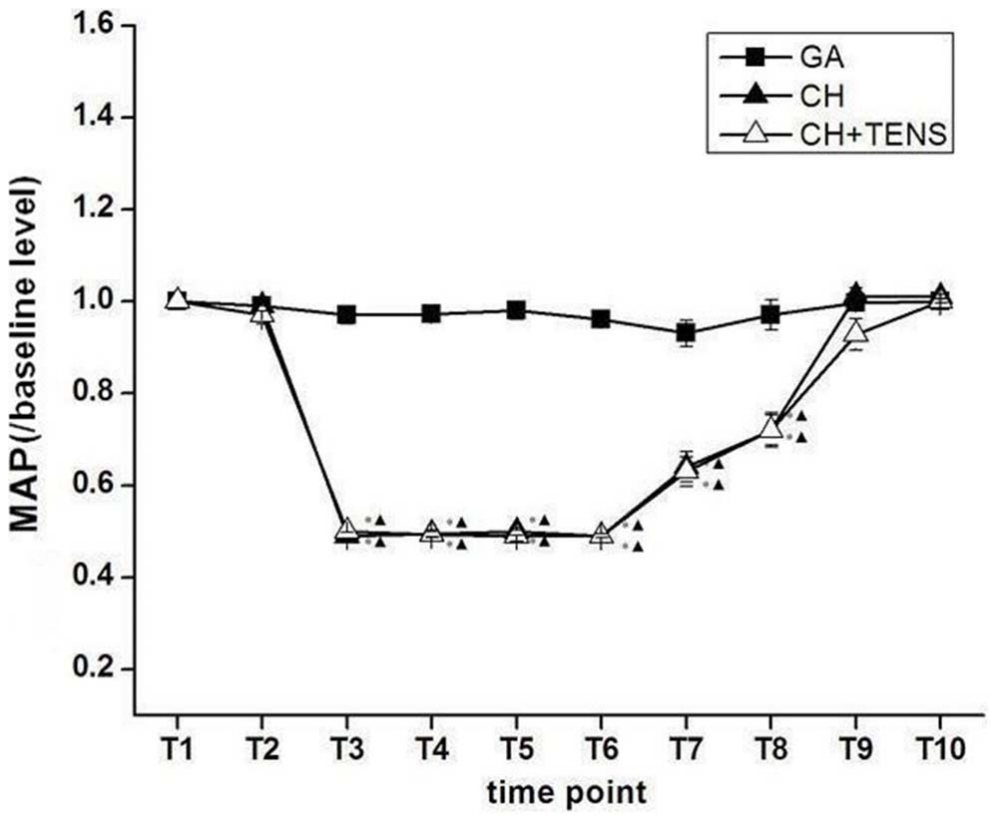
Stability of mean arterial pressure (MAP) levels during controlled hypotension (CH) in all animals. The figure shows that MAP levels in the CH and CH+TENS (2/100 Hz, bidirectional rectangle pulse with 0.6/0.2 ms pulse width,3–5 mA for more than 90 min) groups were kept stable at the target MAP during the maintenance stage of CH (50% of MAP baseline). Data are presented as mean ± standard error of the mean. *P<0.05 vs. general anesthesia (GA) group, ^▴^ *P*<0.05 vs. basal level, n = 6 per group.

MAP levels in all animals were maintained at the same level during necessary anesthetic procedures and before the induction of CH (T_1_–T_2_). MAP in animals from the GA group was maintained at a stable baseline level during the whole experiment. The MAP levels in animals from the two groups administered CH soon reached their target levels, and therefore were decreased significantly (*P*<0.05) compared with baseline MAP and MAP seen in the GA group. During the maintenance stage (T_3_–T_6_) of the target low blood pressure, the MAP levels in the CH and CH+TENS groups were stably sustained at a blood pressure 50% of baseline, which laid the foundation for our follow-up studies. MAP gradually increased after stopping the infusion of SNP (T_6_–T_8_), and blood pressure returned to baseline (T_10_; Figure 2).

### Changes in hepatic blood flow under CH

**Figure 3:**
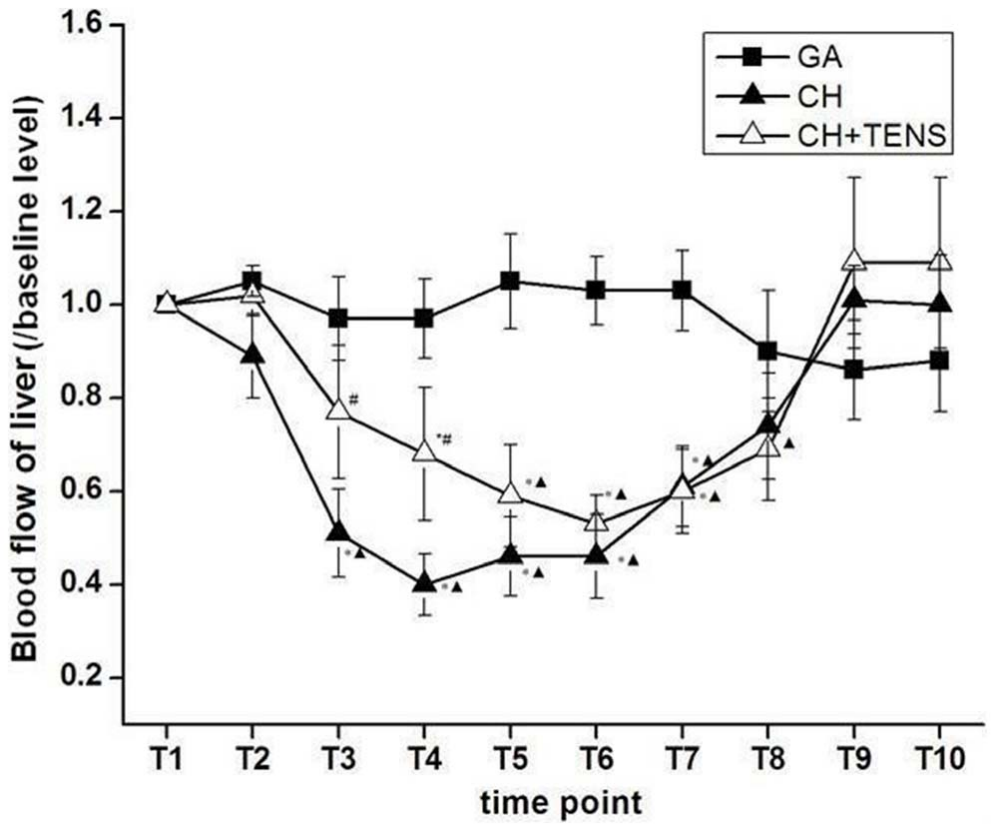
Blood flow to the liver during controlled hypotension (CH). TENS (2/100 Hz, bidirectional rectangle pulse with 0.6/0.2 ms pulse width,3–5 mA for more than 90 min) significantly prevented decreased perfusion to the liver from the beginning of CH to the end of the maintenance stage (T_1_ to T_6_). Data are presented as mean ± standard error of the mean. *P<0.05 vs. GA group, ^▴^ *P*<0.05 vs. basal level, #*P*<0.05 vs. CH group, n = 6 per group.

After anesthesia and before induction of CH (T_1_–T_2_), hepatic blood flow in the CH group began to decline, but did not change in the CH+TENS group. The perfusion of the liver in the CH group was significantly decreased (*P*<0.05) when the target MAP was reached (T_3_), and during the maintenance stage (T_4_–T_6_) when compared with baseline and the GA group. However, the hepatic blood flow in the CH+TENS group only decreased remarkably after the target MAP was maintained for 30–60 min (T_5_–T_6_). In particular, hepatic blood flow was obviously different between the CH and CH+TENS groups in the T_3_–T_4_ period (*P*<0.05). Soon after stopping the infusion of SNP, hepatic blood flow in the two controlled pressure groups gradually (T_7_–T_8_), and finally, completely returned to their respective baseline levels where MAP was maintained at a similar level as the GA group (T_9_–T_10_; Figure 3).

### Changes in renal blood flow under CH

**Figure 4:**
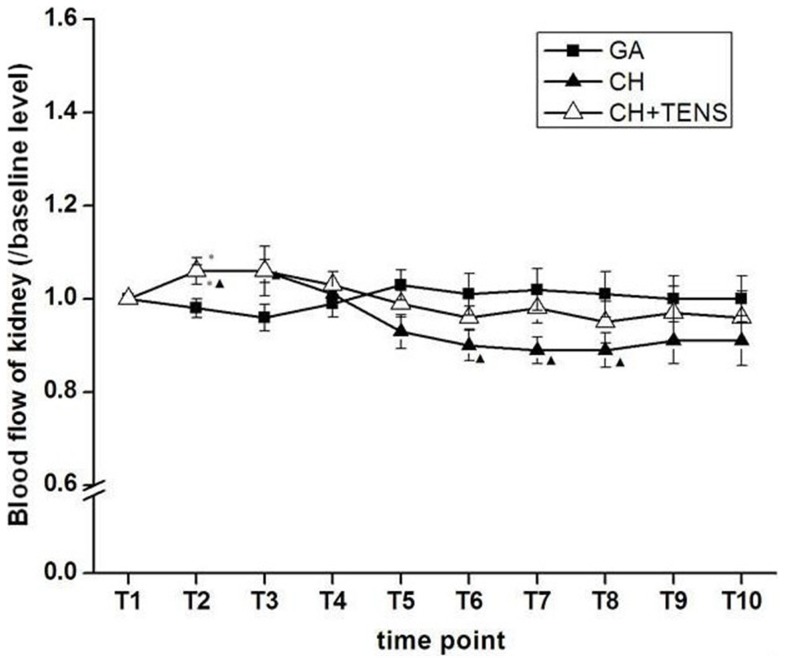
Blood flow to the kidney during controlled hypotension (CH). Renal blood flow in the CH group decreased significantly compared with baseline at the end of the maintenance stage (T_6_) and during MAP restoration (T_7_–T_8_). TENS significantly prevented the low perfusion to the kidney in this period. Data are presented as mean ± standard error of the mean. *P<0.05 vs. general anesthesia (GA) group, ^▴^ *P*<0.05 vs. basal level, n = 6 per group.

During T_2_–T_3_, renal blood flow in the two controlled pressure groups increased significantly (*P*<0.05) when compared with the GA group, instead of decreasing with the decline in MAP. Meanwhile, blood flow to the kidney in the CH+TENS group was maintained at a higher level than baseline (*P*<0.05) during this period. After T_4_, perfusion to the kidney in the two controlled pressure groups began to decline, until the end of the target MAP maintenance stage (T_6_) and the MAP re-establishment phases (T_7_–T_8_). Renal blood flow in the CH group decreased significantly below baseline, however, renal blood flow in the CH+TENS group remained stable and did not decrease during the whole procedure. Additionally, the severity of the reduction in the CH group seemed to be much greater than that of the CH+TENS group. By the end of MAP restoration, renal perfusion in the two CH groups was restored to baseline levels and was maintained at a similar level as the GA group (T_9_–T_10_; Figure 4).

### Changes in gastric blood flow under CH

**Figure 5:**
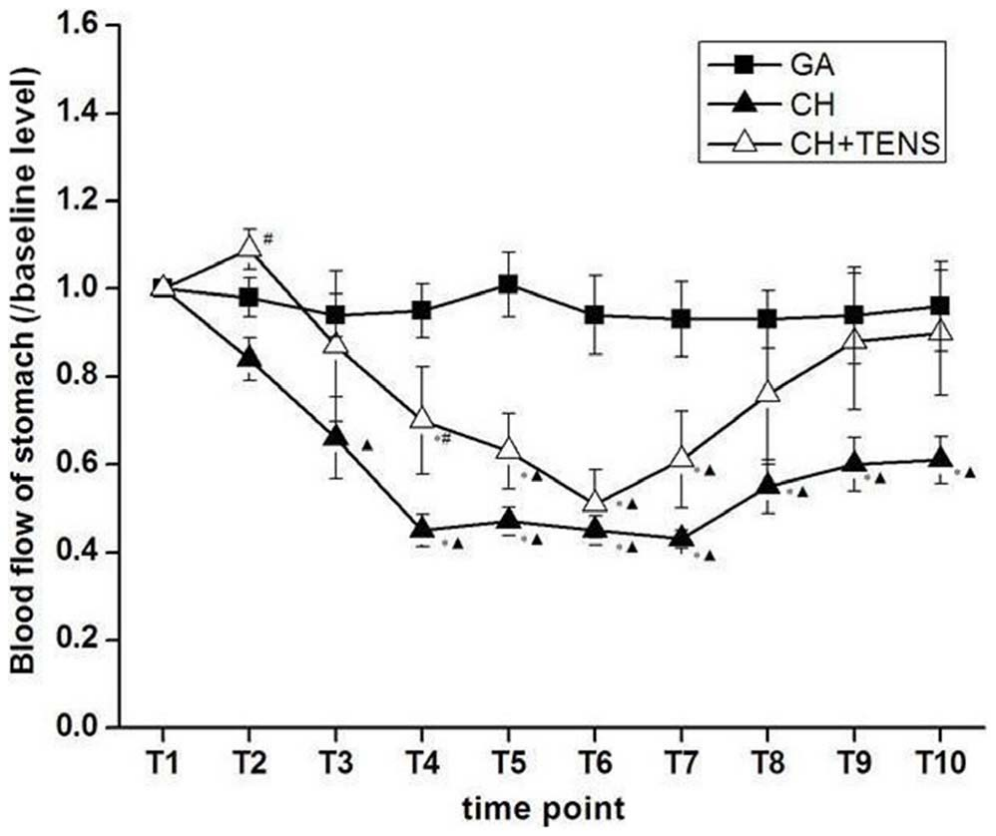
Blood flow to the stomach during controlled hypotension (CH). Gastric blood flow in the CH group was significantly less than baseline levels when mean arterial pressure (MAP) was lowered to reach target pressures and during the first 10 min of maintenance (T_3_–T_4_). TENS significantly prevented the decreased perfusion to the stomach. During the maintenance and MAP restoration stages (T_5_–T_10_), gastric blood flow in the CH group decreased significantly regardless of the cessation of SNP infusion. TENS also effectively inhibited the hypo-perfusion to the stomach in this period. By the end of T_10_, TENS was able to accelerate the recovery of low blood flow to the stomach caused by long-term hypotension. Data are presented as mean ± standard error of the mean. *P<0.05 vs. GA group, ^▴^ *P*<0.05 vs. basal level, #*P*<0.05 vs. CH group, n = 6 per group.

Gastric blood flow in animals from the CH+TENS group increased instead of decreasing with the decline in MAP at the beginning of CH (T_2_). As MAP in the CH group began to decline, gastric blood flow was significantly different between the CH+TENS and CH groups (*P*<0.05). At T_3_–T_4_, gastric blood flow in the CH group was significantly less than baseline (*P*<0.05), however, gastric blood flow in the CH+TENS group did not decrease remarkably, creating a statistical difference between the two groups (*P*<0.05). During T_5_–T_10_, gastric blood flow in the CH group decreased significantly (*P*<0.05) regardless of the cessation of the SNP infusion, while gastric blood flow in the CH+TENS group decreased compared with baseline and the GA group only during T_5_–T_7_. By the end of T_10_, the perfusion to the stomach in the CH+TENS group returned to baseline and equaled the blood pressure seen in the GA group. Gastric perfusion in the CH group did not recover and remained at a low level (Figure 5).

### Changes in cerebral cortical blood flow under CH

**Figure 6:**
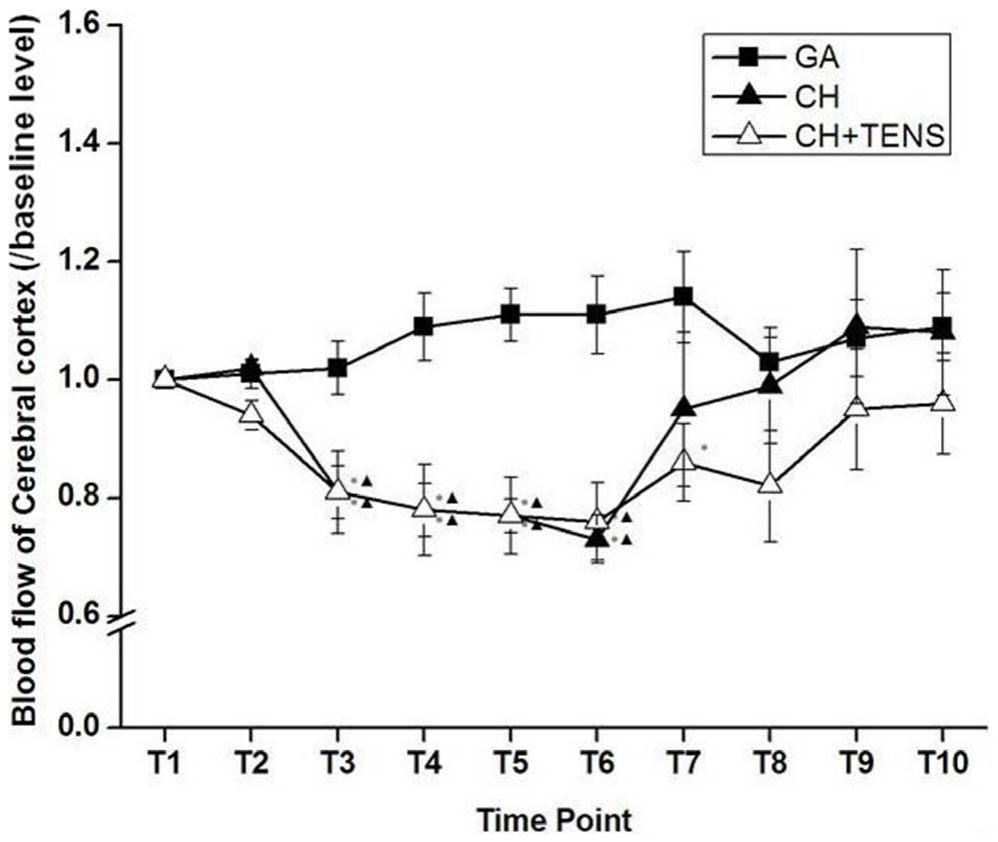
Blood flow to the cerebral cortex during controlled hypotension (CH). During the maintenance stage (T_3_–T_6_), the cerebral cortical blood flow in the CH and CH+TENS (2/100 Hz, bidirectional rectangle pulse with 0.6/0.2 ms pulse width,3–5 mA for more than 90 min) groups decreased significantly compared with their respective baselines and the general anesthesia (GA) group. Soon after stopping the SNP infusion (T_7_–T_10_), cerebral cortical blood flow in the two groups administered CH gradually returned to baseline levels. Given these findings, TENS appeared to have no effect on the perfusion of the cerebral cortex. Data are presented as mean ± standard error of the mean. *P<0.05 vs. general anesthesia (GA) group, ^▴^ *P*<0.05 vs. basal level, n = 6 per group.

During T_1_–T_2_, cerebral cortical blood flow in the CH+TENS group began to decline, while that in the CH group did not change, displaying a contrary pattern of changes compared with hepatic blood flow. During T_3_–T_6_, cerebral cortical blood flow in the two controlled pressure groups decreased significantly compared with baseline and the GA group (*P*<0.05; Figure 5). Soon after stopping the SNP infusion, cerebral cortical blood flow in the CH and CH+TENS groups gradually returned to baseline levels. Additionally, perfusion to the cerebral cortex in the CH+TENS group seemed much less than that in the CH group, however, no significant statistical difference between the two groups was seen (*P*>0.05; Figure 6).

### The occurrence of apoptosis in four major organs after CH

**Figure 7:**
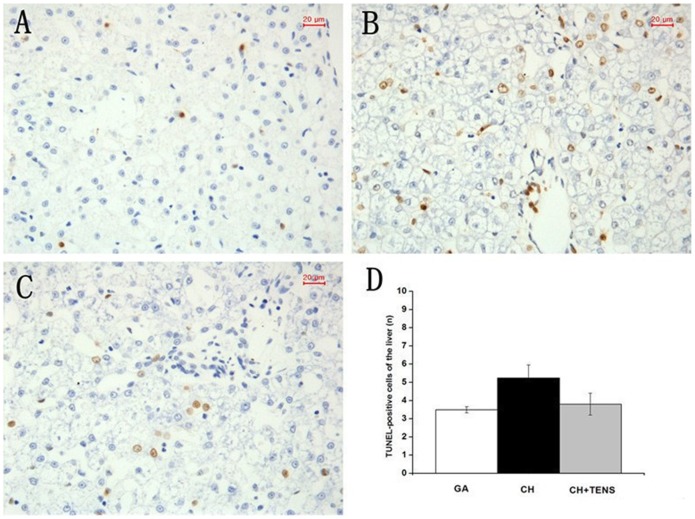
Terminal deoxynucleotidyl transferase-mediated dUTP-biotin nick end labeling (TUNEL) staining of canine hepatic tissue after controlled hypotension (CH). The number of TUNEL-positive cells was quantified at a high magnification (×400). Relatively more TUNEL-positive cells were found in the CH group when compared with the GA group, but this trend was not significant. The number of TUNEL-positive cells is presented as mean ± standard error of the mean. n = 6 per group. Scale bar  = 20 µm.

**Figure 8:**
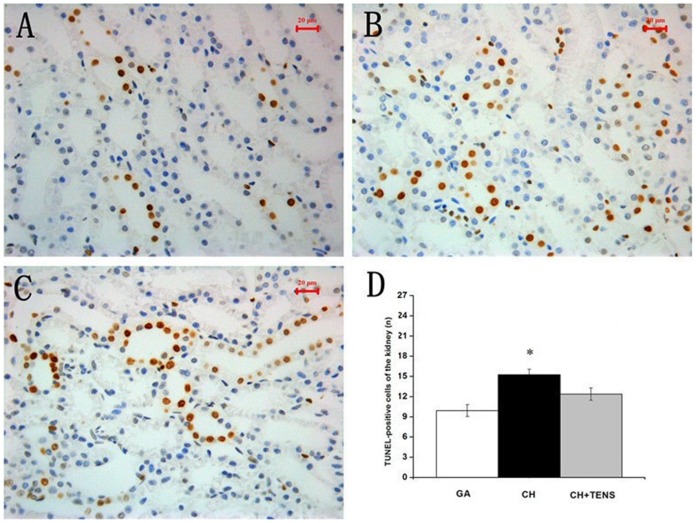
Terminal deoxynucleotidyl transferase-mediated dUTP-biotin nick end labeling (TUNEL) staining of canine renal tissue after controlled hypotension (CH). The number of TUNEL-positive cells was quantified at a high magnification (×400). A significant increase in the number of TUNEL-positive cells was observed in the CH group (B) compared with the GA group (A). The number of TUNEL-positive cells is presented as mean ± standard error of the mean. ^*^ *P*<0.05 vs. GA group, n = 6 per group. Scale bar  = 20 µm.

**Figure 9:**
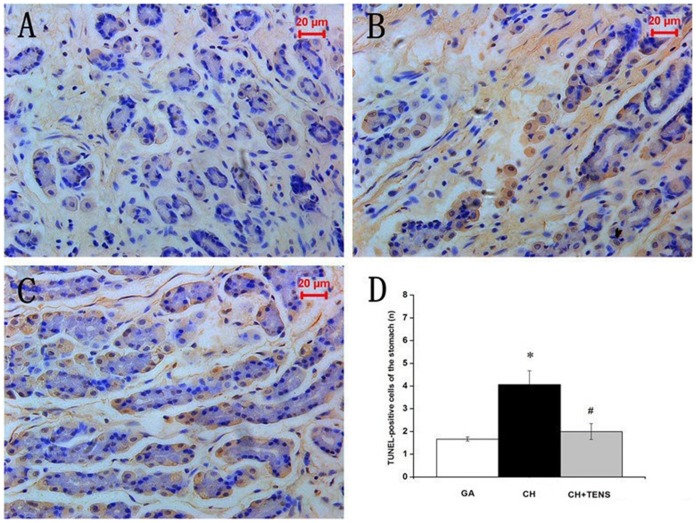
Terminal deoxynucleotidyl transferase-mediated dUTP-biotin nick end labeling (TUNEL) staining of canine gastric mucosa after controlled hypotension (CH). The number of TUNEL-positive cells was quantified at a high magnification (×400). A significant increase in the number of TUNEL-positive cells was seen in the CH group (B) compared with the GA group (A). TENS significantly prevented the increase in TUNEL-positive cells in the TENS group (C) when compared with the GA group (D). The number of TUNEL-positive cells is presented as mean ± standard error of the mean. ^*^ *P*<0.05 vs. GA group, ^#^ *P*<0.05 vs. CH group, n = 6 per group. Scale bar  = 20 µm.

**Figure 10:**
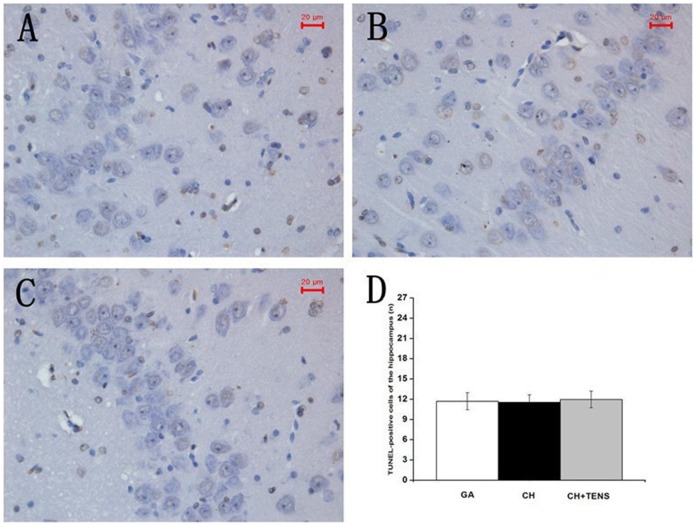
Terminal deoxynucleotidyl transferase-mediated dUTP-biotin nick end labeling (TUNEL) staining of canine hippocampus after controlled hypotension (CH). The number of TUNEL-positive cells was quantified at a high magnification (×400). A stable number of TUNEL-positive cells were found in all three groups (A, B, C), and no significant statistical difference was noted between these groups (D). The number of TUNEL-positive cells is presented as mean ± standard error of the mean. n = 6 per group. Scale bar  = 20 µm.

At 72 h after CH, the segments of the hippocampus, liver, kidney, and stomach were harvested and used for histological examination,and the number of TUNEL-positive cells in the four internal organs was immediately examined. Our results showed that, TUNEL-positive cells in the liver from the CH group tended to increase in number compared with the GA and CH+TENS groups, but this trend was not significantly different (Figure 7). Compared with the GA group, the number of TUNEL-positive cells in the kidney and stomach from animals in the CH group increased significantly (*P*<0.05; Figure 8,9). By contrast, the number of TUNEL-positive cells in these two organs in the CH+TENS group did not increase. Moreover, less TUNEL-positive cells were seen in the stomach from the CH+TENS group compared with the CH group (*P*<0.05; Figure 9). Finally, no changes in the number of TUNEL-positive cells in the hippocampus of animals from all groups were seen after CH (Figure 10).

## Discussion

TENS combined Chinese acupuncture points have been administered as a physical and non-pharmacological therapy for perioperative management of complications caused by surgery [27], [28]. At present, accumulating clinical and animal studies reveal that TENS at acupuncture points, as well as EA treatment, affects hemodynamics and the sympathetic nervous system [19], [29]–[31]. In a recent study, it was suggested that nerve stimulation improves angina pectoris, with a concomitant improvement of myocardial perfusion in cardiac syndrome [32]. Syuu et al. also found EA at the Neiguan (PC 5) acupoint increased stroke volume and cardiac output and further reduced the severity of bleeding-induced hypotension [33], [34]. Dong et al. reported that the changes of perfusion and microcirculation in the stomach were effectively improved after EA given at the Zusanli (ST 36) acupoint [17]. One commonality found in many studies is that TENS has been identified as an effective treatment option to relieve ischemic pain by increasing local blood flow by inducing a vasodilatory effect [16]. In addition, another study also found that TENS can improve the vascular response of aged rats by producing a neurogenic vasodilator effect [35]. From the accumulating evidence, TENS appears to have an analgesic and vasodilating effect that may be causally related.

CH at 50–70% of MAP baseline is routinely performed in clinical practice [1]–[5]. In our previous studies [36], we applied CH at 60% of MAP baseline, and found that the perfusion to the liver and stomach decreased significantly under these conditions, and that apoptosis of major organs did not happen at this level of CH. Considering these facts, we applied CH at 50% MAP in the present study to investigate the perfusion to the liver, kidney, stomach, and cerebral cortex. We also identified whether TENS combined GA played an active role in the redistribution of blood flow and reduction in the occurrence of apoptosis in internal organs.

In this study, before the target MAP for CH was reached, hepatic and gastric perfusion in CH group displayed a similar transient decreasing phenomenon after GA. Conversely, perfusion in the CH+TENS group did not change. Interestingly, cerebral cortical blood flow displayed this transient decreasing phenomenon in the CH+TENS group. These observations support the notion that blood flow to the four investigated vital organs had been redistributed due to GA and TENS intervention for 30 min before CH.

We also found that with a decreasing MAP during CH, blood flows to all four organs decreased by different levels regardless of intervention with TENS, however, the changes in perfusion to each organ were not consistent. During the MAP-lowering stage and early maintenance of target MAP, perfusion to the liver and stomach in the CH+TENS group did not decrease as much as in the CH group, yet the changes in cerebral cortical and renal blood flow were not significantly different. In view of this fact, it can be suggested that TENS had a protective effect on the liver and stomach during this period. During the MAP restoration phase, perfusion to the kidney and stomach in the CH+TENS group was restored more quickly than that in the CH group. By contrast, the rise in MAP, and therefore blood flow to the cerebral cortex and liver, occurred at the same speed in the CH and CH+TENS groups. These observations demonstrate that TENS helped to restore perfusion to the kidney and stomach. It should be noted that when MAP was returning to baseline, hypo-perfusion to the stomach continued in the CH group, but gastric blood flow in the CH+TENS group was restored completely to baseline. Unpredictably, cerebral cortical blood flow in the CH+TENS group was relatively low during the MAP restoration phase, which revealed the possible redistribution of blood flow to vital organs under CH, and how the flow is effected by intervention with TENS. From the changes in blood flow to the vital organs, it could be strikingly observed that the severity of hypo-perfusion to the liver, kidney, and stomach was decreased following CH combined with TENS.

Up until now, studies concerned with apoptosis of the internal organs following TENS or electrical acupuncture stimulation have concentrated on gastrointestinal [37], [38] and cerebral injuries [39]–[42]. Over the past years, many studies have reported that the hippocampus was particularly sensitive to ischemia and hypoxic injury [43] and after transient global ischemia, delayed neuronal death occurred in the CA1 area of the hippocampus [44]. Based on previous research, we regarded apoptosis in the canine hippocampus as an important indicator to detect the condition of cerebral ischemic injury.

By sacrificing dogs 72 h after the application of CH, the number of apoptotic cells in the stomach was significantly less after TENS intervention than that of the CH group. The amount of apoptosis was equivalent to the level seen in the GA group, which was consistent with the changes in gastric blood flow during and after CH. The number of apoptotic cells in the kidney after CH obviously increased, while that in the CH+TENS group after CH did not significantly change, which may be associated with the different renal blood flow seen in the two groups during the target MAP maintenance and MAP restoration stages. Moreover, the number of apoptotic cells in the liver from the CH group was only relatively more than that in the GA group. Considering the hepatic blood flow was restored to baseline by the end of MAP restoration, it could be that temporary lower blood supply in the liver does not induce apoptosis. However, the number of apoptotic cells in the hippocampus from all groups was not significantly different, which was not consistent with the different perfusion levels to the cerebral cortex among the three groups. The reason for this observation is not clear, but might be explained by the cerebral autoregulation of blood flow during hypo-perfusion, which may provide the brain with a stronger self-protecting ability.

### Conclusion

In summary, CH at a 50% MAP level can give rise to the hypo-perfusion of the stomach, liver, kidney, and cerebral cortex in beagle dogs. TENS combined GA can improve the blood supply in the stomach, liver, and kidney to some extent and at different time points. Lower blood flow at this level under CH could cause apoptosis in the stomach and kidney, but not in the liver and hippocampus. TENS administration is able to prevent the apoptosis seen in the stomach and kidney.
